# Identification and Characterization of Two New Degradation Products of Saikosaponin A under Acid Hydrolytic Conditions

**DOI:** 10.3390/molecules21091232

**Published:** 2016-09-14

**Authors:** Jun Li, Qiang Xu, Hua Jiang

**Affiliations:** College of Chemistry and Pharmaceutical Engineering, Henan University of Science and Technology, Luoyang 471023, China; huomanlee@126.com (J.L.); shriib@126.com (Q.X.)

**Keywords:** acid hydrolysis, degradation product, saikosaponin A

## Abstract

Saikosaponin (SS) A is a compound with various pharmacological properties and is easily degraded into SS-B1 and SS-G under acid conditions. In the present work, two new degradation products of SS-A, formed under acid hydrolytic conditions, were detected and isolated using analytical and semi-preparative liquid chromatography technology; furthermore, their structures were characterized as hydroxy-saikosaponin A and SS-B2 by spectral analysis, which is not only essential in stability-indicating method development and validation, but also could be used as a worst case scenario to assess the analytical method performance of SS-A. Moreover, their structural transformation pathways are also proposed.

## 1. Introduction

Radix bupleuri is one of the most important crude drugs in prescriptions of traditional Chinese medicine, which has been utilized to treat influenza, fever, malaria, and menstrual disorders for approximately 2000 years. According to the Chinese Pharmacopoeia, radix bupleuri consists of the roots of *Bupleurum chinense* DC. and *B. scorzonerifolium* Willd. In addition to the official species, there are approximately 40 species of the *Bupleurum* grown in China, such as *B. marginatum* var. *stenophyllum*, *B. komarovianum*, and *B. yinchowense*, among which the species *Bupleurum falcatum* L. is mainly distributed in Japan [1,2,3,4,5].

**Figure 1:**
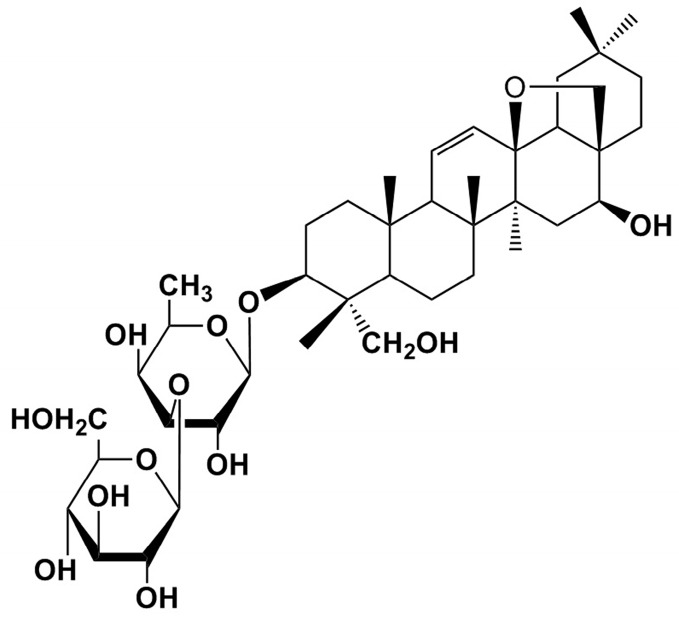
Structural formula of SS-A.

SS-A (Figure 1) is a major bioactive compound in the herbal medicines of radix bupleuri and exhibits various pharmacological properties, such as being an anticonvulsant, an antiepileptic, and anti-inflammatory [6,7,8]. Recent research has paid attention to its anti-inflammatory effects in different physiological processes [8,9,10]. Studies have proposed several signaling pathways in inflammatory response after treatment of SS-A, including cyclooxygenase-2 (COX-2), inducible nitric oxide synthase (iNOS), transcription factor nuclear factor kappa B (NF-κB), toll-like receptor 4 (TLR4), and mitogen-activated protein kinase (MAPK) [5,9,10,11]. Thus, SS-A possesses the potential to be a drug candidate, which originates from natural products, to clinically treat inflammation.

During the development of SS-A as a drug product, the International Conference on Harmonization of Technical Requirements for Registration of Pharmaceuticals for Human Use (ICH) guidelines mention the necessity of stress testing in order to elucidate the inherent stability of a drug substance and the characterization of all degradation products formed by drug products. The information obtained may help in the establishment of degradation patterns and a better quality control method [12,13].

Previous research has indicated that, among all the stress conditions mentioned in the ICH guidelines including heating in water, dry heating, oxidation, exposure of alkali solutions and light, SS-A was relatively stable, except in the case of stress under acid hydrolytic condition, in which H^+^ serves as an effective accelerator of the degradation process. A thorough literature survey revealed that the compounds SS-B1 and SS-G have been well documented as the degradation products of SS-A under acid conditions [14,15,16,17,18]. However, in our detailed studies on the forced degradation of SS-A under acid hydrolytic conditions using the optimized chromatographic method, we found four total degradation compound peaks, which indicated the existence of two new degradation products. In accordance with ICH regulatory guidelines, the two new degradation products of SS-A needed to be characterized, which would not only help to identify the conditions under which additional controls should be employed in manufacturing and storage, but would also be useful in the prediction or experimental determination of the toxicity of the identified degradation products [19].

The present manuscript describes the structural characterization of the two new degradation products of SS-A, under acid hydrolytic conditions, and their structural transformation pathways. 

## 2. Results and Discussion

### 2.1. Identification of New Acid Degradation Products

**Figure 2:**
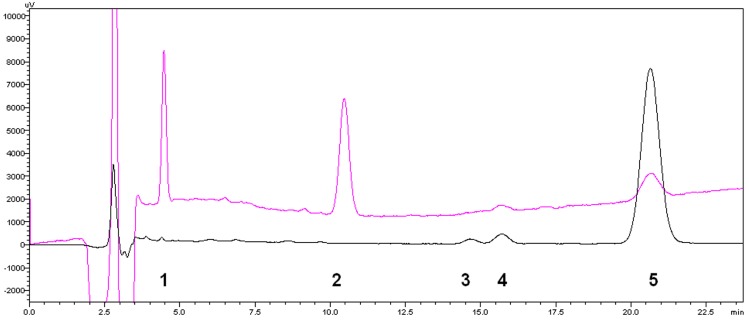
HPLC chromatography of SS-A (**2**) and its acid degradation products (**1**, **3**, **4**, **5**) under the detection wavelengths of 210 nm (**pink line**) and 254 nm (**black line**).

The HPLC method was employed to achieve the best separation of the acid hydrolysis sample of SS-A. During the optimization process, preliminary studies were carried out on a Hypersil C18 column (4.6 mm × 250 mm, 5 mm) using a combination of methanol and H_2_O (70:30, *v*/*v*) as the mobile phase [20]. However, the peaks corresponding to the degradation products were not completely resolved. Peak 1 (Figure 2) was mixed with the solvent peaks at 210 nm, and Peaks 3 and 4 (Figure 2) were badly overlapped at 254 nm. To get an acceptable separation between SS-A and its degradation products, the flow rate and composition of the mobile phase were systematically varied to optimize the method [21]. Finally, an adequate separation of peaks, with a good resolution, was obtained using methanol (A) and H_2_O (B) in a gradient mode. The solvent program was set as follows: *T*_min_/A:B; *T*0/40:60; *T*8.0/40:60; *T*10/50:50; *T*20/40:60; and *T*26/40:60. A flow rate of 1.0 mL·min^−1^, a column temperature at 30 °C, an injection volume of 20 μL, and a wavelength of 210 and 254 nm were found to be suitable for achieving separation (Figure 2).

Under optimized HPLC conditions, four degradation compound peaks were easily found (Figure 2). However, a literature search revealed that, under acidic conditions, there were only two degradation products reported for SS-A, which were SS-B1 and SS-G [14,15,16,17,18]. Therefore, there were another two new degradation products of SS-A that had not been clarified. Using retention time comparison experiments with the authentic substances, peaks with retention times of 21.50 min and 16.00 min were identified as SS-B1 and SS-G, respectively. Then, the peaks with retention times of 4.50 min and 14.75 min were recognized as two, new, unknown degradation products of SS-A (Table 1).

### 2.2. Characterization of the Two New Degradation Compounds 

The two new degradation compounds, arising from SS-A under the stress of acid hydrolysis, led to interest regarding the evolution of its structure, which would not only be useful to the establishment of a stability-indicating method for SS-A as a potential drug candidate, but could also provide more clues in the new understanding of the structure transformation mechanism. The isolation was performed on a semi-preparative HPLC system, and NMR spectral analyses were used for structure evaluation.

#### 2.2.1. Degradation Compound of Peak 1

**Figure 3:**
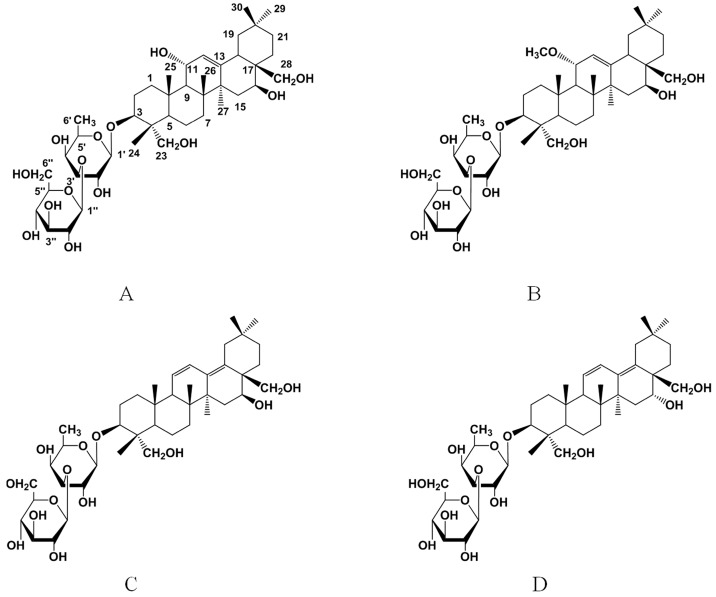
Structural formulas of hydroxy-saikosaponin A (**A**); SS-B3 (**B**); SS-B1 (**C**); and SS-B2 (**D**).

Peak 1 was isolated as a white amorphous powder, a positive Molisch reaction, of which the molecular formula was determined to be C_42_H_70_O_14_ by the pseudomolecular ion peak at *m*/*z* 821.4546 [M + Na]^+^ in the positive HRESI-MS (High Resolution Electrospray Ionization Mass Spectrometry) experiment. In the UV spectrum, there is no absorption peak, except for a slope curve from 220 nm to 200 nm (Supplementary Materials Figure S2), which indicated the absence of conjugated double bonds [15]. The ^13^C-NMR spectrum was similar to that of SS-B3 (Table 2) [22,23]. However, the signal of a methoxyl group was detected in SS-B3, but not in Peak 1. By comparing the ^13^C-NMRchemical shifts of Peak 1 with those of SS-B3, the C-11 signal of the aglycone shifted upfield by 9.1 ppm, and the neighboring C-9 and C-12 signals shifted downfield by 1.7 ppm and 5.6 ppm (Table 2), respectively. These spectral data suggest that Peak 1 possesses a hydroxyl group at C-11 of the aglycone moiety, but not a methoxyl group as in SS-B3. Thus, its structure was determined as de-11-*O*-methylsaikosaponin B3 and was named hydroxy-saikosaponin A (Figure 3). NMR data were in agreement with previously reported literature [24,25].

#### 2.2.2. Degradation Compound of Peak 3

Peak 3 was obtained as a white amorphous powder, a positive Molisch reaction, and exhibited a pseudomolecular ion peak in its positive-ion HRESI-MS at *m*/*z* 803.4559 [M + Na]^+^, corresponding to the molecular formula of C_42_H_68_O_13_. The UV spectrum shows characteristic heteroannular diene absorption (λ_max_ 242, 254, 263 nm) (Supplementary Materials Figure S6) [15]. The ^13^C-NMR spectroscopic data of Peak 3 showed a close resemblance to those of SS-B1 (Table 2) [22,23]. The main difference was the signal at C16, which was shifted upfield by 8.6 ppm in Peak 3. The spectral data indicated that an α-OH was located at C16 of the aglycone in Peak 3, while a β-OH was linked at C16 in SS-B1. Then, the structure of Peak 3 was decided to be SS-B2 (Figure 3). NMR data were identical to what has been reported in the literature [24,26].

### 2.3. Structural Transformation Mechanism of Hydroxy-Saikosaponin A

**Figure 4:**
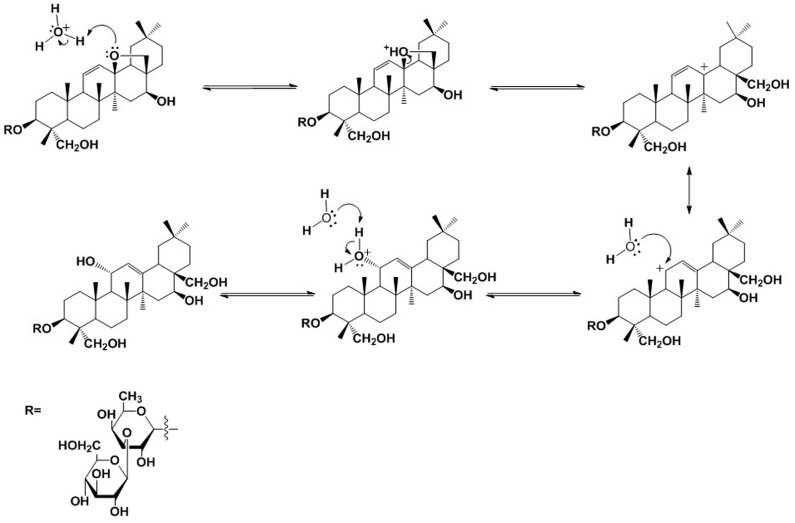
Proposed structural transformation pathway of hydroxy-saikosaponin A.

SS-B1 was reported as the main degradation product of SS-A; however, there is little literature recognizing hydroxy-saikosaponin A as a degradation product derived from SS-A under the acid hydrolysis process. Figure 4 shows the proposed structural transformation route of hydroxy-saikosaponin A. Firstly, a proton transfers from the hydronium ion (H_3_O^+^) to the O atom of the 13β-28 epoxy ring in SS-A, forming an alkyloxonium ion, where a dissociation is involved giving an allylic carbocation at C13. Because a vinyl group is an extremely effective electron-releasing substituent, a resonance interaction of the type shown permits the π electrons of the double bond at C11 and C12 to be delocalized dispersing the positive charge, which means that the positive charge is shared by the two end carbons in the allylic unit. The positive charge of the carbon, and the vacant *p* orbital, combine to make carbocation strongly electrophilic, which could readily react with H_2_O, which serves as a nucleophile. Additionally, because of the steric hindrance, H_2_O would more likely react with the carbonation at C11 than that at C13. The bonds to the positively charged carbon are coplanar and define a plane of symmetry in carbocation, which is achiral. Theoretically, H_2_O would attack the carbocation at its two mirror-image faces, yielding two alkyloxonium ions with different configurations. However, taking the effect of steric hindrance into consideration, the alkyloxonium ion with an α relative configuration at C11 predominates. Finally, another H_2_O acts as a base to remove a proton from the alkyloxonium ion in order to give the product of hydroxy-saikosaponin A.

### 2.4. Structural Transformation Mechanism of SS-B2

**Figure 5:**
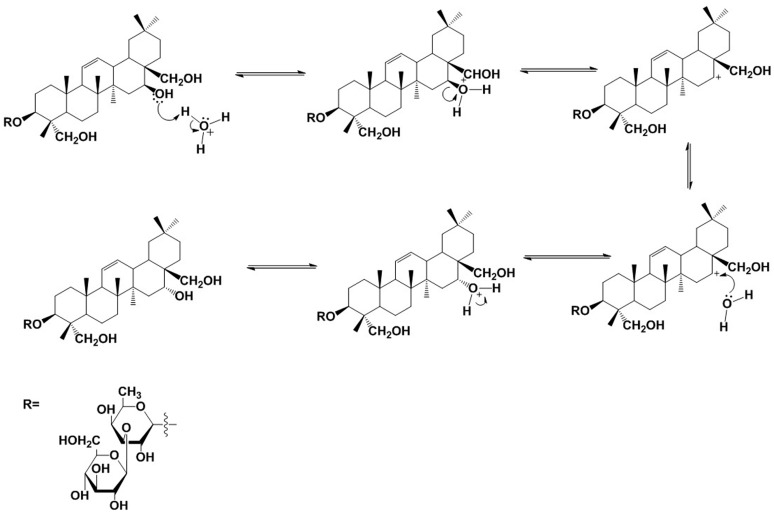
Proposed structural transformation pathway of saikosaponin B2.

SS-B2 was known as a degradation product of SS-D, an isomer of SS-A, with the only difference being the configuration of OH at C16. It was first detected in the acid hydrolysis system of SS-A in our study. Based on in-depth observation of the structural relationship, SS-B2 was proposed to be derived from the compound SS-B1. Being similar to hydroxy-saikosaponin A, the formation of SS-B2 is described in Figure 5. Protonation of the O atom of the hydroxyl group at C16 takes place, mediated by the hydronium ion (H_3_O^+^) forming an alkyloxonium ion, which was followed by a dissociation that gives a molecule of H_2_O and a carbocation at C16. Then, another H_2_O acts as a nucleophile to capture the carbocation from the direction, with a low level of steric hindrance, which permits the alkyloxonium ion at C16 with an α relative configuration. Then, the deprotonation of the alkyloxonium ion yields the product of SS-B2, during which H_2_O acts as a Brønsted base.

### 2.5. Structural Transformation Path of SS-A under Acid Conditions

**Figure 6:**
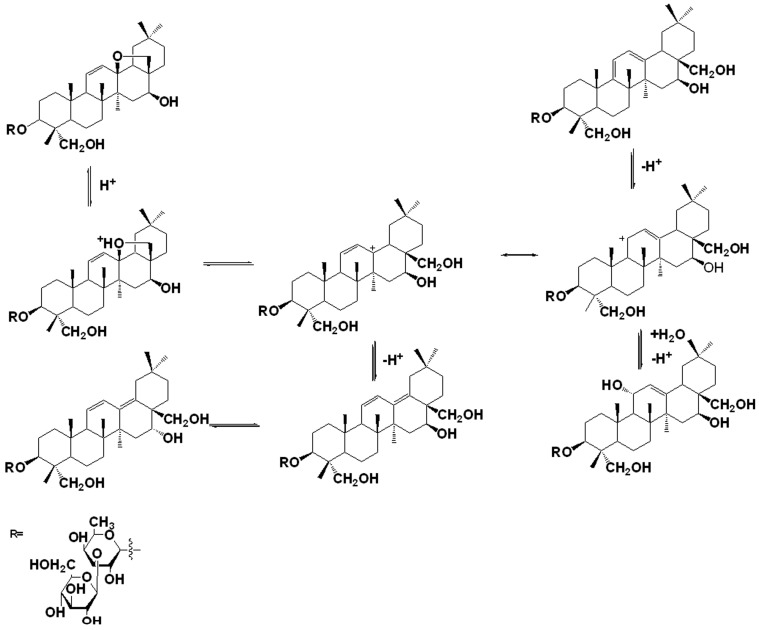
Structural transformation map of saikosaponin A under the stress of acid hydrolytic conditions.

In acid conditions, SS-A could decompose not only to the known degradation compounds, SS-B1 and SS-G, but also to SS-B2 and hydroxy-saikosaponin A, which were detected as two new degradation compounds. It is clear that SS-B2, SS-B1, and SS-G are the isomers of SS-A, and hydroxy-saikosaponin A is derived from the hydration reaction of SS-A with H_2_O. The structural transformation path of SS-A under acid condition is displayed in Figure 6.

Moreover, in acidic conditions, SS-A could also be hydrolyzed into prosaikogenin F, prosaikogenin A, prosaikogenin H, saikogenin F, saikogenin A, and saikogenin H, where the hydrolysis reaction takes place on the glycosidic bond of the sugar chain [15]. However, in our study, the above-mentioned aglycones and monofucoside of SS-A were not detected, which could be explained by the fact that it is difficult for the hydrolysis reaction to take place on the sugar side chain under the reaction conditions used in this research.

## 3. Materials and Methods

### 3.1. Chemicals and Reagents

SS-A, SS-B1, and SS-G were supplied by Yuanye Bio-Technology Co., Ltd. (Shanghai, China). Methanol (HPLC grade) was purchased from Dikma (Tianjin, China) and used without further purification. Analytical reagent grade hydrochloric acid and sodium hydroxide pellets were purchased from Wulian Chemical Factory (Shanghai, China). Ultrapure water was obtained from Milli-Q water (18M) (Millipore, Bedford, MA, USA). All other chemicals were analytical reagent.

### 3.2. Preparation of Standards and Sample Solutions

#### 3.2.1. Standard Solutions

SS-A, SS-B1, and SS-G were separately weighed into three 10-mL volumetric flasks and dissolved in methanol to obtain stock solutions of 200 μg/mL.

#### 3.2.2. Sample Solutions

SS-A was dissolved in water with the aid of heat and sonication. The stock was diluted to 50:50 (*v*/*v*) with 0.2N HCl, which was heated at 80 °C for 10 h. After cooling, the solution was neutralized by using a NaOH solution and applied into a glass column (20 mm × 300 mm, i.d.) packed with AB-8 macroporous resin (Chemical Plant of Nankai University, Tianjin, China). Deionized water was used to remove the NaCl, and 70% aqueous ethanol was used to elute the target compounds. After eluting, solvent was removed using a rotary evaporator under vacuum, and the residue was lyophilized. Three milligrams of the lyophilized sample was redissolved in 10 mL of methanol and filtered through 0.45-μm membrane filters for HPLC analysis. In addition, 100 mg of sample was used for the further purification of the degradation compounds in a semi-preparative HPLC.

### 3.3. HPLC Analysis

The chromatographic separation of compounds was performed on a Shimadzu Prominence LC-20A System (Shimadzu, Kyoto, Japan) equipped with a degasser (DGU-20A5), a binary gradient pump (LC-20AB), and a diode array detection system (SPD-M20A, Shimadzu). The column used was a 5-μm Hypersil ODS C18 column (4.6 mm × 250 mm, i.d.), which was operated at a temperature of 30 °C. Methanol (A)-H_2_O (B) was used as the mobile phase. The LC gradient program was set as (*T*_min_/A:B; *T*0/40:60; *T*8.0/40:60; *T*10/50:50; *T*20/40:60; *T*26/40:60). The flow rate was 1.0 mL·min^−1^. The injection volume was 20 μL, and the wavelengths were set at 210 nm and 254 nm.

### 3.4. Isolation by Semi-Preparative HPLC

Purified degradation compounds of SS-A, for structural evaluation using spectra analyses were isolated from the extract by means of repeated semi-preparative HPLC (Chuangxin Tongheng Co. Ltd., Beijing, China). The column used was a YMC-Pack ODS-A Column (5 μm, 20 mm × 250 mm, i.d.). The mobile phase was (*T*min/A:B; *T*0/30:70; *T*20/40:60; *T*40/35:65; *T*50/30:70) with detection wavelengths of 210 and 254 nm. The flow rate was set at 5.0 mL·min^−1^, and the injection volume was 500 μL. The purity of the compounds was examined by analytical HPLC–DAD (DiodeArray Detector, Shimadzu).

### 3.5. NMR Spectra

NMR spectra were recorded on a Bruker AV-400 spectrometer (Bruker, Zug, Switzerland, using C_5_D_5_N as solvent. Tetramethylsilane (TMS) was used as an internal standard. The chemical shifts (δ) are in ppm and the coupling constants (*J*) are in Hz. The sample for NMR analysis was obtained using semi-preparative HPLC (LC3000, Chuangxin Tongheng Co. Ltd.).

## 4. Conclusions

According to the ICH guidelines on impurities, the identification of degradation products formed under a variety of stress conditions as well as the outlining of degradation pathways and mechanisms are required. The two new degradation products formed during the forced degradation study of SS-A under the stress of acid hydrolysis conditions were identified as SS-B2 and hydroxy-saikosaponin A, which are not only essential in stability-indicating method development and validation, but can also be used as a worst-case scenario to assess the analytical method’s performance for SS-A. Moreover, it can also help to identify conditions under which additional controls should be employed in manufacturing and storage. It is also very valuable to understand the reactive chemistry of SS-A, with respect to the Quality by Design (QbD) knowledge space. Furthermore, it is useful to the establishment of mass balance at an early stage during forced degradation studies and prevents later surprises during formal stability studies [19].

## Figures and Tables

**Table 1:** Degradation products of saikosaponin A formed in acid-forced degradation experiments.

No.	Peak Label	Retention Time (min)	Peak Information
1	Peak-1	4.50	Unknown impurity
2	Peak-2	11.50	Saikosaponin A
3	Peak-3	14.75	Unknown impurity
4	Peak-4	16.00	Saikosaponin G
5	Peak-5	21.50	Saikosaponin B1

**Table 2:** ^13^C-NMR spectral data comparison of Peak 1 with SS-B3 and Peak 3 with SS-B1 (C_5_D_5_N, δppm).

No.	Peak1	SS-B3	Peak3	SS-B1	No.	Peak1	SS-B3	Peak3	SS-B1
1	41.5	40.1	38.4	38.4	23	64.3	64.2	64.7	64.0
2	26.8	26.5	26.1	26.1	24	13.7	13.6	13.1	13.1
3	81.8	81.7	81.6	81.5	25	18.3	18.2	18.4	18.9
4	43.9	43.9	43.8	43.7	26	18.3	18.5	17.4	17.0
5	47.8	47.6	47.3	47.3	27	26.8	26.5	21.8	22.0
6	17.8	17.9	18.9	18.2	28	68.7	68.5	64.7	64.0
7	33.3	33.3	32.5	32.6	29	33.5	33.3	25.1	24.9
8	41.0	41.0	41.2	40.5	30	24.0	24.0	32.3	32.4
9	55.7	54.0	54.0	54.5	CH_3_O		52.1		
10	36.9	36.8	36.7	36.5	1′	106.1	106.0	106.0	106.0
11	66.8	75.9	126.3	127.0	2′	71.6	71.8	71.8	71.7
12	128.1	122.5	126.3	125.7	3′	85.2	85.3	85.5	85.4
13	145.2	148.3	136.2	136.4	4′	71.9	71.9	71.9	71.7
14	43.9	43.9	42.0	44.2	5′	71.1	71.0	71.1	71.1
15	38.2	37.0	32.6	35.0	6′	17.4	17.2	17.2	17.2
16	66.4	66.2	67.9	76.5	1′′	106.8	106.7	106.7	106.7
17	43.9	44.0	45.4	44.4	2′′	75.9	75.9	75.9	75.8
18	43.9	44.0	133.1	133.3	3′′	78.9	78.8	78.7	78.8
19	46.5	47.0	39.1	38.4	4′′	72.2	72.2	72.2	72.2
20	31.0	31.1	32.7	32.6	5′′	78.5	78.5	78.5	78.8
21	34.2	34.2	35.6	35.1	6′′	62.8	62.7	62.9	62.7
22	26.0	26.2	24.6	30.0					

## Supplementary Materials

Supplementary materials can be accessed at: http://www.mdpi.com/1420-3049/21/9/1232/s1.
