# Supplementary material for: Identification and Characterization of Two New Degradation Products of Saikosaponin A under Acid Hydrolytic Conditions
Source: Molecules. 2016 Sep 14;21(9):1232. doi: 10.3390/molecules21091232 (PMC6274035; doi:10.3390/molecules21091232)
Supplement: Supplementary file 1 [file molecules-21-01232-s001.pdf]

# Supplementary Materials: Identification and Characterization of Two New Degradation Products of Saikosaponin A under Acid Hydrolytic Conditions

**Jun Li, Qiang Xu and Jiang Hua**

MS Formula Results: + Scan (8.264 min) Sub (2014102903.d)

| m/z | Ion | Formula | Abundance |
|---|---|---|---|
| 821.4546 | (M+Na)+ | C42 H70 Na O14 | 130068 |

| Best | Formula (M) | Ion Formula | Score | Cross Sco | Mass | Calc Mass | Calc m/z | Diff (ppm) | Abs Diff (ppm) | Mass Match | Abund Match | Spacing Match | DBE |
|---|---|---|---|---|---|---|---|---|---|---|---|---|---|
| ☑ | C42 H70 O14 | C42 H70 Na O14 | 99.86 | | 798.4698 | 798.4711 | 821.4603 | 1.65 | 1.65 | 99.91 | 99.75 | 99.89 | 9 |

**Figure S1.** HRESI-MS data for hydroxy-saikosaponin A.

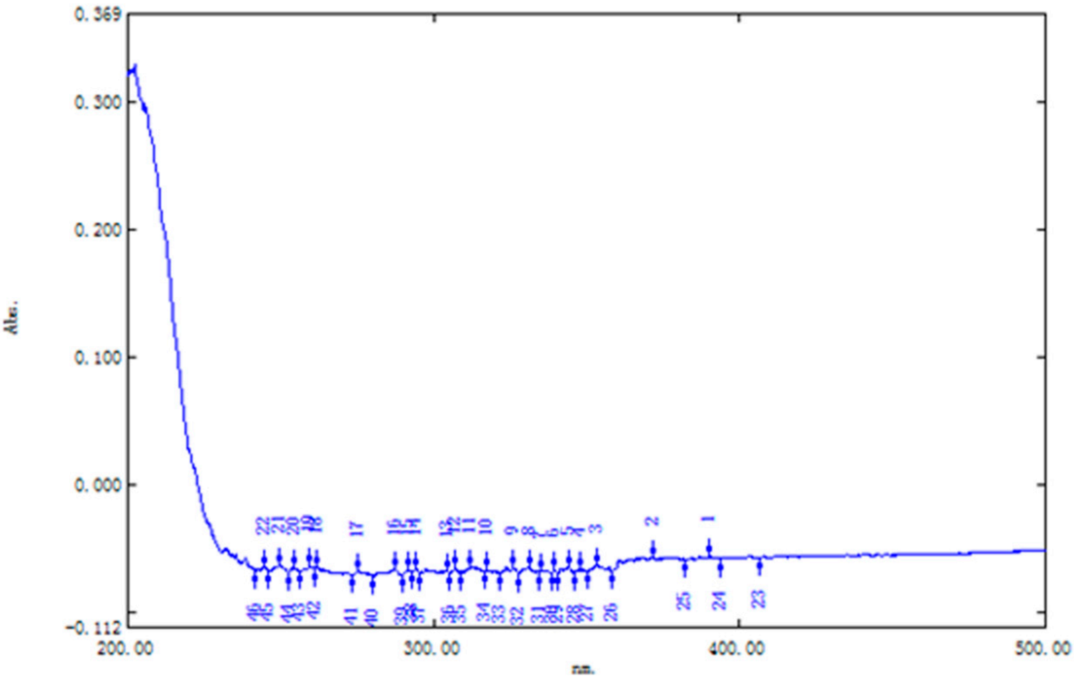


**Figure S2.** UV spectrum of hydroxy-saikosaponin A.

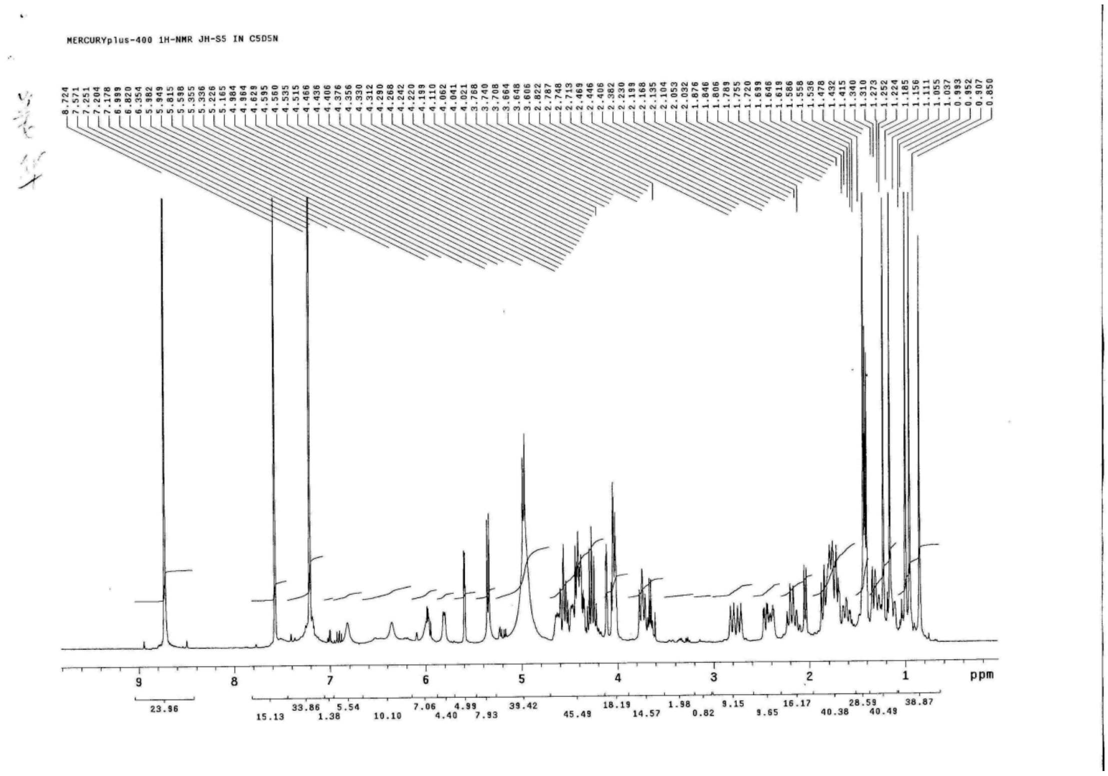

**Figure S3.** $^1$H-NMR spectrum of hydroxy-saikosaponin A.

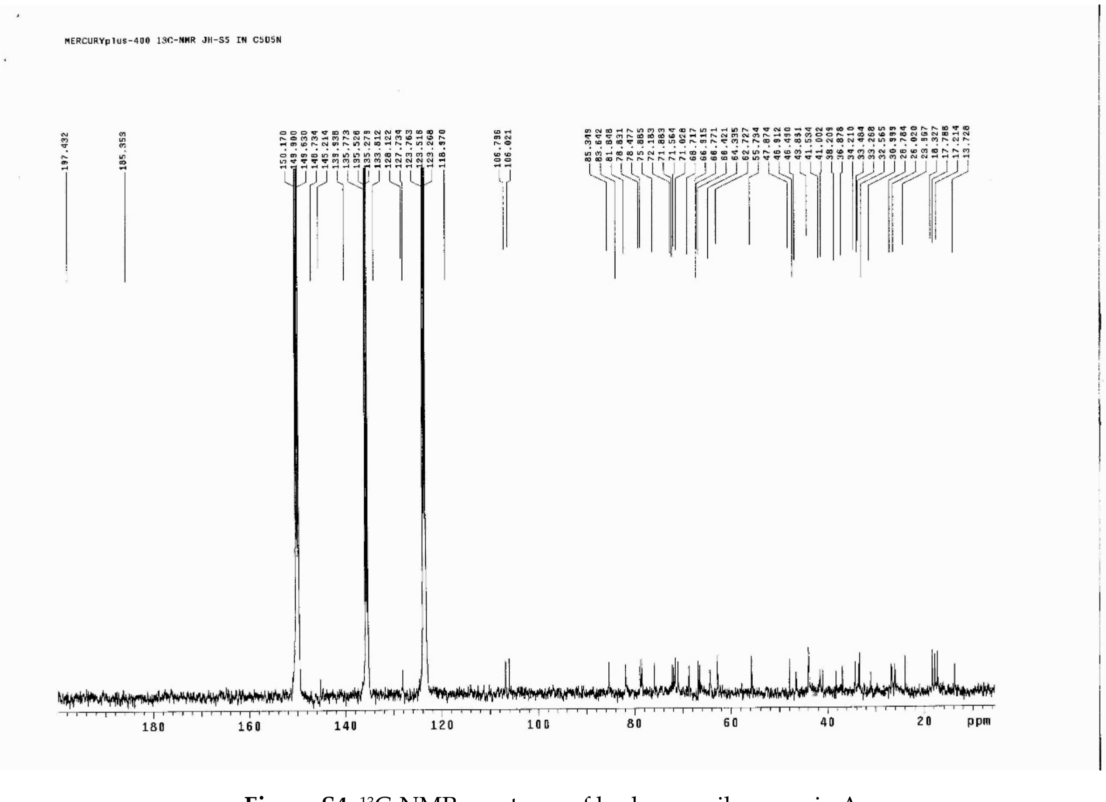

**Figure S4.** $^{13}$C-NMR spectrum of hydroxy-saikosaponin A.

MS Formula Results: + Scan (8.231 min) Sub (2014102901.d)

| m/z | Ion | Formula | Abundance |
|---|---|---|---|
| 803.4559 | (M+Na)+ | C42 H68 Na O13 | 1085403.5 |

| Best | Formula (M) | Ion Formula | Score | Cross Sco | Mass | Calc Mass | Calc m/z | Diff (ppm) | Abs Diff (ppm) | Mass Match | Abund Match | Spaci |
|---|---|---|---|---|---|---|---|---|---|---|---|---|
| ☑ | C42 H68 O13 | C42 H68 Na O13 | 99.91 | | 780.4667 | 780.466 | 803.4552 | -0.97 | 0.97 | 99.97 | 99.76 | |

**Figure S5.** HRESI-MS data of saikosaponin B2.

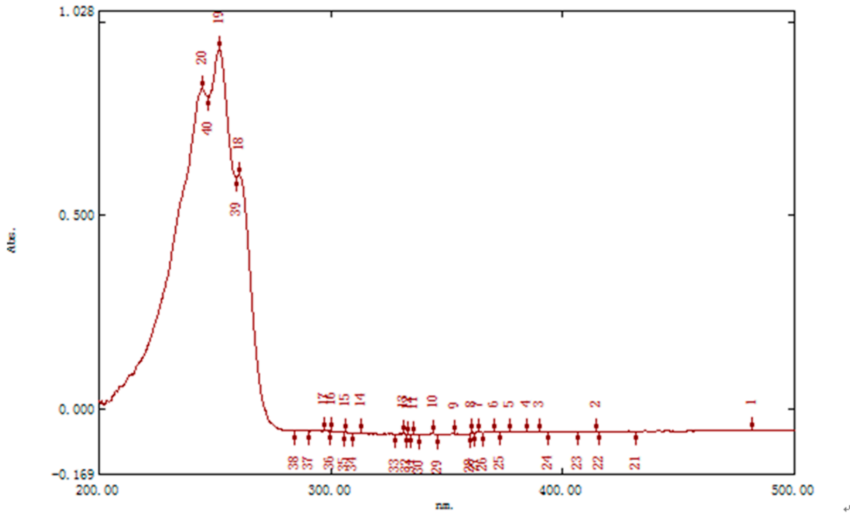


**Figure S6.** UV spectrum of saikosaponin B2.

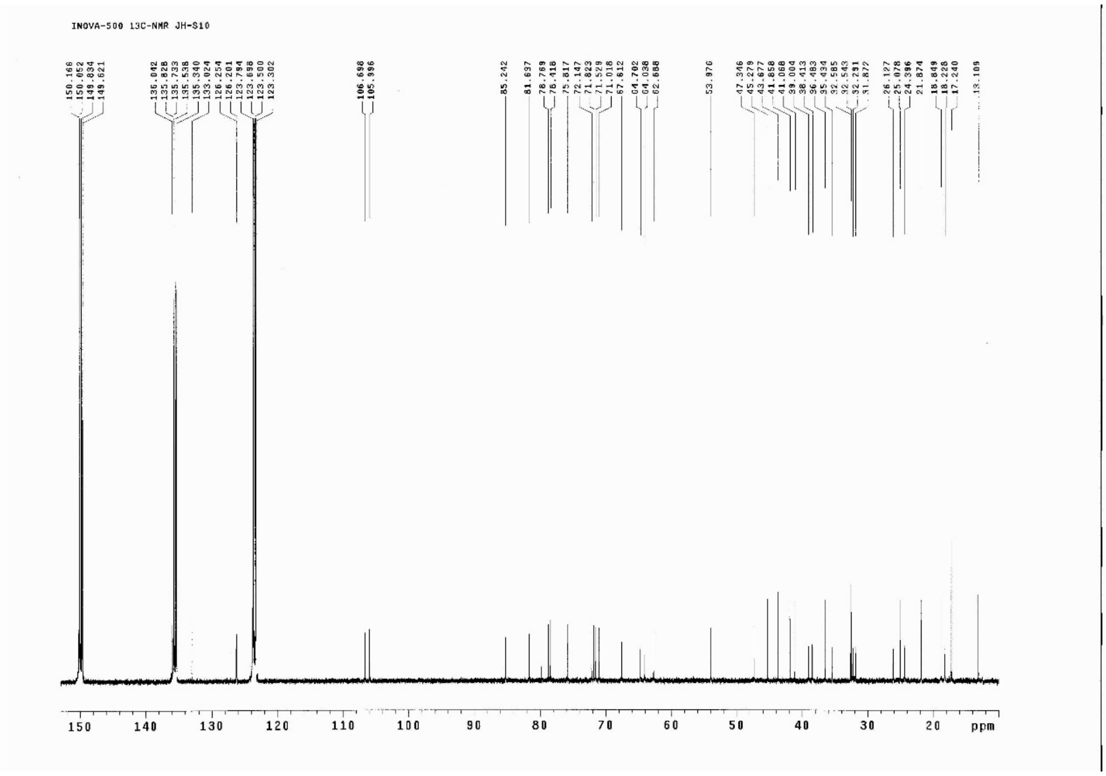


**Figure S7.** $^{13}$C-NMR spectrum of saikosaponin B2.

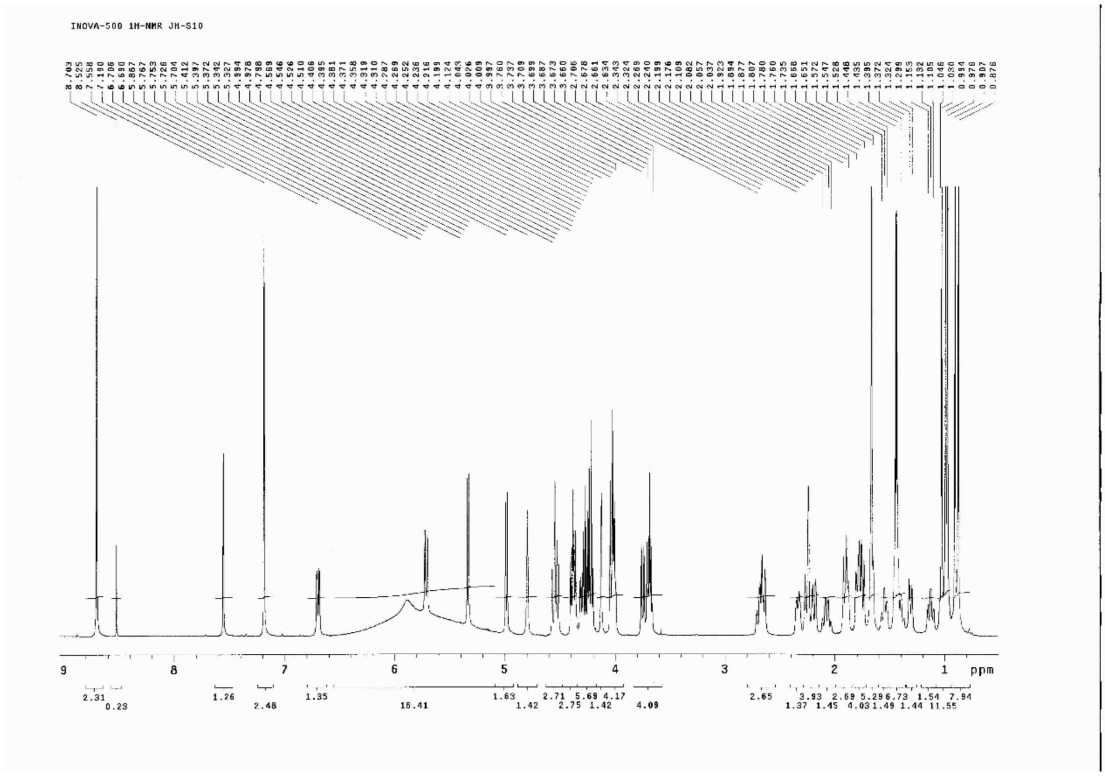


**Figure S8.** $^{1}$H-NMR spectrum of saikosaponin B2.
